# Genomewide characterization of non-polyadenylated RNAs

**DOI:** 10.1186/gb-2011-12-2-r16

**Published:** 2011-02-16

**Authors:** Li Yang, Michael O Duff, Brenton R Graveley, Gordon G Carmichael, Ling-Ling Chen

**Affiliations:** 1 Department of Genetics and Developmental Biology, University of Connecticut Stem Cell Institute, University of Connecticut Health Center, 263 Farmington Ave, Farmington, CT 06030-6403, USA; 2 State Key Laboratory of Molecular Biology, Institute of Biochemistry and Cell Biology, Shanghai Institutes for Biological Sciences, Chinese Academy of Sciences, 320 Yue Yang Road, Shanghai 200031, PR China; 3 Current address: Shanghai Institutes for Biological Sciences, Chinese Academy of Sciences, 320 Yue Yang Road, Shanghai 200031, PR China

## Abstract

**Background:**

RNAs can be physically classified into poly(A)+ or poly(A)- transcripts according to the presence or absence of a poly(A) tail at their 3' ends. Current deep sequencing approaches largely depend on the enrichment of transcripts with a poly(A) tail, and therefore offer little insight into the nature and expression of transcripts that lack poly(A) tails.

**Results:**

We have used deep sequencing to explore the repertoire of both poly(A)+ and poly(A)- RNAs from HeLa cells and H9 human embryonic stem cells (hESCs). Using stringent criteria, we found that while the majority of transcripts are poly(A)+, a significant portion of transcripts are either poly(A)- or bimorphic, being found in both the poly(A)+ and poly(A)- populations. Further analyses revealed that many mRNAs may not contain classical long poly(A) tails and such messages are overrepresented in specific functional categories. In addition, we surprisingly found that a few excised introns accumulate in cells and thus constitute a new class of non-polyadenylated long non-coding RNAs. Finally, we have identified a specific subset of poly(A)- histone mRNAs, including two histone H1 variants, that are expressed in undifferentiated hESCs and are rapidly diminished upon differentiation; further, these same histone genes are induced upon reprogramming of fibroblasts to induced pluripotent stem cells.

**Conclusions:**

We offer a rich source of data that allows a deeper exploration of the poly(A)- landscape of the eukaryotic transcriptome. The approach we present here also applies to the analysis of the poly(A)- transcriptomes of other organisms.

## Background

Nascent pre-mRNA transcripts undergo multiple co-transcriptional/post-transcriptional processing and modification events during their maturation. A poly(A) tail is added post-transcriptionally to the 3' end of almost all eukaryotic mRNAs and plays an important role in mRNA stability, nucleocytoplasmic export, and translation [1]. 3' end formation involves binding of the cleavage/polyadenylation machinery to the AAUAAA hexamer (or some variants), often together with a downstream G/U rich sequence, followed by endonucleolytic cleavage of the pre-mRNA and the addition of a 3' non-templated poly(A) tail of up to 200 to 250 adenosines in mammalian cells [2]. As most known mRNAs are polyadenylated at their 3' ends, transcriptome analysis using deep sequencing (mRNA-seq) typically involves enrichment of poly(A)+ RNAs by oligo(dT) selection [3-6]. However, this approach precludes detection of transcripts lacking a poly(A) tail.

A number of functional long transcripts (defined here as those >200 nucleotides in length) are known to lack poly(A) tails. These non-polyadenylated transcripts (poly(A)- RNAs) include ribosomal RNAs (rRNAs) generated by RNA polymerase I and III, other small RNAs generated by RNA polymerase III, and replication-dependent histone mRNAs [7] and a few recently described long non-coding RNAs (lncRNAs) [8,9] synthesized by RNA polymerase II. Unlike poly(A)+ RNAs, the 3' end processing mechanisms of poly(A)- transcripts are quite distinct from each other. While most histone pre-mRNAs contain evolutionarily conserved stem-loop structures in their 3' UTRs that direct U7 small nuclear RNA (snRNA)-mediated 3' end formation [7], the lncRNAs *malat1 *and *menβ *are processed at their 3' ends by RNase P (which also processes the 5' ends of tRNAs), but also both encode a highly conserved short poly(A) tract at their 3' ends [8,9].

Apart from histone mRNAs and the other transcripts mentioned above, relatively little is known about poly(A)- transcripts or mRNAs with short poly(A) tails. Earlier evidence suggested the existence of non-histone polysomal-associated poly(A)- RNAs [10,11], but these were not characterized in detail. In addition, Katinakis *et al. *[12] suggested that some transcripts can be 'bimorphic' and exist in both poly(A)+ and poly(A)- forms, and that bimorphic ones can be produced from poly(A)+ RNAs that are processed to reduce or totally remove the poly(A) tail under certain conditions. This observation was further supported by more recent studies. By searching for the conserved poly(A)-limiting element, Gu *et al. *[13] identified several hundred sequences in human cells that possess poly(A) tails of <20 nucleotides. By separating RNAs into two fractions depending on the length of their poly(A) tails (short and long poly(A) tails) followed by a microarray analysis, Meijer *et al. *[14] found that approximately 25% of expressed genes have a short poly(A) tail of less than 30 residues in a significant percentage of their transcripts in NIH3T3 cells. The larger scale bioinformatic studies also suggested that a significant fraction (>24%) of long non-coding transcripts present in cells may lack a classical poly(A) tail [15-17]. Cheng *et al. *[15] used tiling arrays to detect total RNAs from ten human chromosomes in multiple human cell lines and Wu *et al. *[16] used 454 sequencing to characterize the 3' ends of transcripts regardless of whether or not they contained a poly(A) tail. Both groups identified many long poly(A)- transcripts, though there was relatively little overlap between the poly(A)- transcripts identified in these two studies.

In the current study, we have used deep sequencing to separately characterize the poly(A)+ and poly(A)- enriched transcriptomes from both HeLa cells and H9 human embryonic stem cells (hESCs). By comparing the relative abundance of long transcripts (>200 nucleotides) in the poly(A)- and the poly(A)+ libraries, we have identified populations of bimorphic and poly(A)- transcripts. These transcripts include not only known long poly(A)- transcripts such as histone mRNAs, precursors for Cajal body related small RNAs, and lncRNAs, but many other non-polyadenylated (or short poly(A)-tail-containing) transcripts of protein-coding genes and intron-derived lncRNAs. We also observed that some replication-dependent histone mRNAs are specifically expressed in pluripotent cells, and thus may constitute a unique group of markers for pluripotency.

## Results and discussion

### Identification of poly(A)- transcripts by RNA-Seq

**Figure 1:**
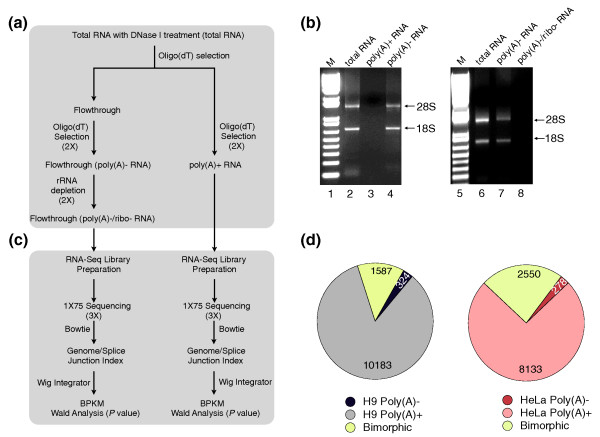
**Poly(A)+, poly(A)- and bimorphic transcripts revealed by deep sequencing**. **(a) **A diagram of the experimental approach. Total RNAs were extracted from H9 cells or HeLa cells and treated with DNaseI before being subjected to poly(A)+ and poly(A)- transcript enrichment. See text for details. The enriched poly(A)- and poly(A)+ RNAs were used to prepare single-end RNA-Seq libraries. The size-selected single-end libraries were sequenced using 76 cycles. The single-end reads were trimmed from the 3' end to a total length of 75 nucleotides prior to alignment. **(b) **Agarose gel electrophoresis to confirm the poly(A)- RNA purification. The gel on the left shows that the poly(A)+ RNA fraction from HeLa cells contains no detectable rRNA but that the poly(A)- material not bound to oligo(dT) beads contained most of the rRNA. The gel on the right shows that subsequent rRNA depletion removes the great majority of rRNA from the poly(A)- sample. M, the molecular weight marker. **(c) **A diagram of the analytical approach. Sequence analysis involved aligning all reads to a combined database of the genome and splice junctions using Bowtie [15,19]. The read counts were then further analyzed using the normalized value BPKM (bases per kilobase of gene model per million mapped bases) to identify poly(A)- and bimorphic transcripts that were significantly different between the poly(A)+ and poly(A)- samples. **(d) **Classification of poly(A)+, poly(A)- and bimorphic predominant transcripts. Poly(A)+, poly(A)- and bimorphic predominant transcripts were classified according to their relative abundance between the poly(A)+ and poly(A)- samples in individual cell lines. See text and Materials and methods for details.

Library preparation for typical RNA-seq experiments begins with oligo(dT) selection to enrich for poly(A)+ RNAs or with rRNA depletion to enrich for non-rRNAs. In this study, we enriched for poly(A)- transcripts by keeping the unbound fraction from multiple rounds of oligo(dT) selection, followed by two rounds of rRNA depletion. As a control, poly(A)+ RNAs were also collected using oligo(dT) selection (Figure 1a,b). These two RNA populations were prepared from both H9 hESCs and HeLa cells from which RNA-Seq libraries were generated by performing RNA fragmentation, random hexamer primed cDNA synthesis, linker ligation and PCR enrichment. Size selection (Materials and methods) allowed us to enrich for long transcripts.

All libraries were then sequenced in three lanes on the Illumina Genome Analyzer II*x *(GAII*x*) platform. Since the correlation among lanes was greater than r^2 ^= 0.98 (Additional file 1), we the combined data from all lanes of each sample to obtain between 37 and 54 million 75-nucleotide reads from each library (Additional file 2). We used Bowtie [18,19] to align the reads to a combined database of the *Homo sapiens *genome (GRCh37/hg19) and annotated splice junction sequences. Figure 1c shows a diagram of our analytical approach. For the poly(A)- libraries, approximately 5.0 and 6.0 million reads in H9 cells and HeLa cells were uniquely aligned, respectively, compared with approximately 23.0 and 33.4 million reads from poly(A)+ samples in H9 cells and HeLa cells (Additional file 2).

We used the uniquely aligned reads to determine the extent of the genome covered by at least 1 or 2 reads. We found that 3.3% and 3.8% of the genome was mapped by at least one read in the H9 and HeLa poly(A)- samples, respectively (Additional file 2), while 0.8% and 1.2% of the genome was mapped by at least two reads in the H9 and HeLa poly(A)- samples, respectively. In contrast, in the poly(A)+ samples, 5.5% and 6.8% of the genome were mapped with at least one read (Additional file 2) and 2.4% and 3.2% of the genome were mapped with at least two reads in H9 and HeLa cells, respectively. Note that due to performing rRNA depletion, size selection, and unique mapping, our poly(A)- data did not include rRNAs, abundant short RNAs (microRNAs, piwi-interacting RNAs (piRNAs), and small interfering RNAs (siRNAs)), tRNAs, snRNAs, many small nucleolar RNAs (snoRNAs) and repetitive transcripts such as the abundant *Alu *elements, long interspersed nuclear elements (LINEs) and endogenous long terminal repeats.

### Classification of poly(A)+, poly(A)-, and bimorphic transcripts

We next classified all expressed annotated transcripts as being either poly(A)+, poly(A)-, or bimorphic predominant subgroups according to their relative abundance using BPKM (bases per kilobase of gene model per million mapped bases; see Materials and methods and [20]) values for each gene in the poly(A)+ and poly(A)- samples from the same cell line (Figure 1d). Poly(A)- predominant transcripts (for simplicity we use the term 'poly(A)- transcripts' throughout this study) were defined as those with BPKM ≥1, *P *< 0.05 and at least two-fold greater enrichment from the poly(A)- library compared to the poly(A)+ library. In contrast, poly(A)+ predominant transcripts ('poly(A)+ transcripts') were defined as those with BPKM ≥1, *P *< 0.05 and at least two-fold greater enrichment from the poly(A)+ library compared to the poly(A)- library. Bimorphic-predominant transcripts ('bimorphic transcripts') were defined as those with BPKM ≥1, *P *< 0.05 and less than two-fold relative expression between the poly(A)+ and poly(A)- libraries (Figure 1d). A number of apparently poly(A)- or bimorphic genes were discarded following manual examination because they had low/inconsistent expression patterns or contained alternative transcripts expressed from introns. For example, WDR74 was originally identified as a poly(A)- transcript, but the processed WDR74 mRNA is poly(A)+. Mis-characterization resulted from very high expression of an intronic poly(A)- small RNA. Thus, we removed WDR74 from the poly(A)- list. Using the above criteria, we found that although most (84.2% in H9 cells and 74.2% in HeLa cells) of the annotated expressed transcripts are poly(A)+, a significant portion of genes (13.1% in H9 cells and 23.3% in HeLa cells) are bimorphic. In addition, 2.7% and 2.5% of the annotated transcripts are poly(A)- in H9 and HeLa cells, respectively (Figure 1d). Full gene lists are available in Additional files 3 and 4.

It has previously been estimated that between 60% and 80% of transcripts are either poly(A)- or bimorphic [15,16], a significantly higher number than what we observed. This could be due to numerous technical and experimental differences between the previous studies and ours.

### Validation of poly(A)- and poly(A)+ transcripts

**Figure 2:**
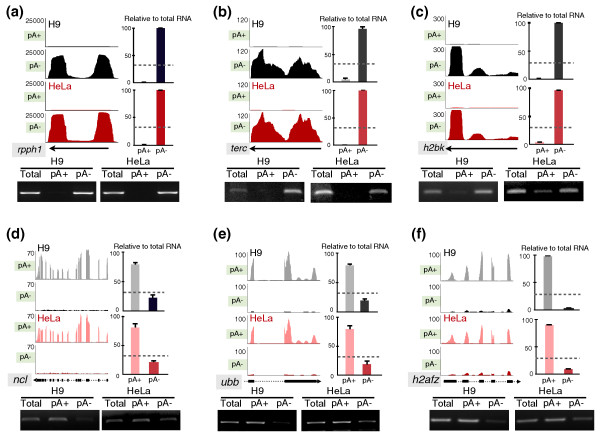
**Validation of selected poly(A)+ and poly(A)- transcripts**. **(a-c) **Validation of known poly(A)- transcripts. Y-axis: normalized read densities of each gene from the UCSC genome browser (left panels). Quantitative RT-PCR (qRT-PCR) was performed with independent poly(A)+ and poly(A)- sample preparations, and the relative signals from each enriched RNA preparation were normalized to those in the total RNA preparations from different cell lines (right panels). Note that the signals for poly(A)- transcripts were significantly enriched in the poly(A)- samples. **(d-e) **Validation of known poly(A)+ transcripts. Normalized read densities (left panels) and qRT-PCRs (right panels) were analyzed as described above. Note that the signals for poly(A)+ transcripts were significantly enriched in the poly(A)+ samples. Grey, poly(A)+ sample from H9 cells; black, poly(A)- sample from H9 cells; pink, poly(A)+ sample from HeLa cells; red, poly(A)- sample from HeLa cells. Dashed lines, the cutoff ratio used to assign poly(A)+ and poly(A)- transcripts (the abundance in either poly(A)+ or poly(A)- fractionation accounts for more than one-third when compared to the total RNA). Gene models are shown beneath the UCSC genome browser screenshots. See text for details. These descriptions are also used for other figures throughout this study. Error bars were calculated from three biological repeats.

To further validate the approach we used to enrich poly(A)- RNAs and to demonstrate that our criteria for poly(A)- and bimorphic classifications accurately reflect transcripts expressed in human cells, we used both semi-quantitative PCR and real-time PCR (qPCR) to examine the relative distribution of a number of known polyadenylated and non-polyadenylated RNAs in the poly(A)+ and poly(A)- populations from both cell lines (Additional files 5 and 6). For poly(A)- RNAs, we selected *rpph1*, the RNA component of RNase P, *terc*, the RNA component of telomerase, and *hist1h2bk *(*histone cluster 1*, *h2bk*), which encodes a histone transcript known to lack a poly(A) tail. As expected, in our sequence data, we only observed *rpph1*, *terc *and *hist1h2bk *in the poly(A)- samples in both cell lines (Figure 2a-c, black and red colors). Semi-quantitative RT-PCR and qPCR confirmed that 95 to 99% of these transcripts are in the poly(A)- fraction (Figure 2a-c), validating our poly(A)- RNA isolation procedure. This conclusion was further strengthened by the distribution of other non-polyadenylated lncRNAs, *malat1 *and *neat1 *(its long isoform, also called *menbeta *in mouse), in our sequence data. Each of these contains a genomically encoded conserved poly(A) tract positioned at the 3' end of the transcript followed by an RNase P processing site [8,9] (Additional file 7). The pattern of coverage across the *malat1 *lncRNA was unexpectedly different in the poly(A)+ and poly(A)- datasets (Additional file 7a). Coverage of *malat1 *is highly enriched at the 3' end of the transcripts in the poly(A)- fraction yet relatively uniform in the poly(A)+ fraction. While we do not yet know the basis for this phenomenon, it is apparent both in HeLa cells and H9 cells. Examination of the relative abundance of different regions of *malat1 *by semi-quantitative PCR showed that different regions of this lncRNA were equally abundant in poly(A)- samples (Additional file 7b). Taken together, these results lead us to suggest that the 5' ends of the poly(A)- isoforms of *malat1 *are being degraded slowly or that the 5' region is modified somehow so that it cannot be aligned to the genome.

We next examined several transcripts that are known to contain a poly(A) tail. These included *ncl *(*nucleolin*), *ubb *(*ubiquitin *B) and *h2afz *(*h2a histone family*, *member z*). These mRNAs were enriched in the sequence data from the polyA(+) samples for both cell lines (Figure 2d-f, grey and pink colors). As expected, semi-quantitative RT-PCR and qRT-PCR confirmed that 80 to 90% of these mRNAs were present in the poly(A)+ samples in both H9 cells and HeLa cells (Figure 2d-f). In addition, one known polyadenylated lncRNA, the short isoform of *neat1 *[9,21], was also significantly enriched in the poly(A)+ sample from HeLa cells (Additional file 7c,d), validation data not shown). Taken together, these validation experiments demonstrated that our method can successfully identify poly(A)+ and poly(A)- transcripts, allowing for a thorough analysis of the transcriptome, including RNAs with different types of 3' ends.

### Characterization of bimorphic transcripts

**Figure 3:**
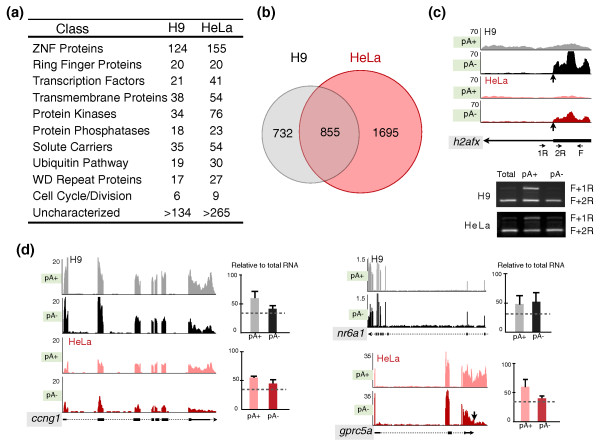
**Classification of bimorphic transcripts**. **(a) **Gene ontology analysis of bimorphic transcripts according to their functions; see text for details. **(b) **Overlapping analysis of the expression of bimorphic transcripts in H9 and HeLa cells. **(c) **An example of a bimorphic histone mRNA, *hist1h2afx*. Normalized read densities of *hist1h2afx *from the UCSC genome browser (upper panels). Note that the two isoforms were distinct in poly(A)+ and poly(A)- samples from H9 and HeLa cells. Bottom panels, semi-quantitative RT-PCR with primers that recognize either the longer poly(A)+ transcript or both transcripts confirmed the observations from the deep sequencing. F, forward primer; 1R and 2R, reverse primers. The vertical arrow depicts the position of U7-mediated 3' end formation. **(d) **Validation of identified bimorphic transcripts, *ccng1*(left panels), *nr6a1 *(right upper panels) and *gprc5a *(right bottom panels). Normalized read densities and qRT-PCRs were analyzed as described above. Note that the signals for these transcripts were similar in both poly(A)+ and poly(A)-samples. See text for details. Error bars were calculated from three biological repeats.

Bimorphic transcripts are those that do not clearly fall into either the poly(A)+ or poly(A)- categories. Some of these may result from poly(A)+ RNAs that are processed to reduce or totally remove their poly(A) tails under certain conditions [12]. These RNAs do not efficiently bind to oligo(dT) beads under our experimental conditions and therefore should be detected in both poly(A)+ and poly(A)- samples. We thus classified the RNAs that are present at similar levels (less than two-fold) in both the poly(A)+ and poly(A)- libraries as bimorphic RNAs. We identified 2,550 and 1,587 bimorphic RNAs from HeLa and H9 cells, respectively, accounting for 23.3% of the expressed transcripts in HeLa cells and 13.1% in H9 cells (Additional files 8 and 9). Gene ontology analysis revealed that mRNAs encoding members of zinc finger (ZNF) proteins, ring finger proteins, transcription factors, transmembrane proteins, protein kinases, protein phosphatases, solute carriers, ubiquitin pathway, WD repeat proteins, cell cycle, and a number of functionally uncharacterized transcripts were overrepresented in the bimorphic group in both cell lines (Figure 3a). Interestingly, more than half of the identified bimorphic transcripts from H9 cells were also expressed and classified as bimorphic in HeLa cells, indicating that the bimorphic nature we observed for these transcripts was reproducible (Figure 3b; Additional file 10). For instance, *h2afx *(*h2a histone family*, *member x*), the only known bimorphic histone transcript, is bimorphic in our analysis (Figure 3c, upper panel). Notably, the shorter isoform (processed by U7-mediated cleavage at its 3' end [22,23]; Additional file 11) showed significant enrichment in the poly(A)- samples (black and red) in both H9 and HeLa cells, while the longer isoform containing a poly(A) tail was largely detected only in the poly(A)+ samples. Semi-quantitative PCR confirmed these observations. Primers that selectively amplify the poly(A)+ transcripts yielded a product only in the poly(A)+ samples, while primers that amplify both poly(A)+ and poly(A)- transcripts yielded products in both RNA samples (Figure 3c, bottom panel).

We next randomly selected several bimorphic mRNAs that are expressed either in both cell types (*cyclin G1*, *ccng1*), uniquely in H9 cells (*nuclear receptor subfamily 6*, *group A*, *member 1*, *nr6a1*), or uniquely in HeLa cells (*G protein-coupled receptor*, *family C*, *group 5*, *member A*, *gprc5a*) (Additional files 8 and 9), and performed real time RT-PCR to confirm their relative abundance in both RNA fractions. The results confirmed that each of the tested transcripts is present at comparable levels in both the poly(A)+ and poly(A)- samples (Figure 3d). It will be of interest to further investigate whether there are common structural features or sequence motifs that regulate the length of the poly(A) tail in these transcripts. For example, studies by Gu *et al. *[13] indicated that the poly(A)-limiting element is a conserved *cis*-acting sequence that can regulate poly(A) tail length. Several hundred sequences with poly(A) tails of <20 nucleotides were found in human cells, and, consistent with the results of our gene ontology analysis, an extended family of ZNF transcription factors were overrepresented in this list [13]. Owing to a lack of precision of the precise 3'-processing sites of many of our bimorphic transcripts (they do not match the annotated ends), it is not yet possible to compare our results directly with those of Gu *et al. *[13].

In addition, as we classify transcripts according to their ability to bind to oligo(dT) cellulose, we cannot discriminate the truly bimorphic transcripts, such as *h2afx *and *neat1*, from those whose poly(A) tails are shortened during normal transcript metabolism. While it is not clear exactly how long a tail is necessary for retention on oligo(dT), or how long mRNAs persist once their tails are shortened, in our experiments, many of these transcripts behave in the same way (low affinity to oligo(dT)) in both cell lines, and *h2afx *and *neat1 *are accurately classified as bimorphic transcripts under our selective standards. On the other hand, it is possible that some transcripts may have encoded A stretches that might result in retention on oligo(dT) to some extent. We therefore examined some known mRNAs of this type. The conserved human repetitive *Alu *elements contain long A stretches, and *Alu *elements are embedded in the 3' UTRs of many transcripts, such as *nicn1*, *paics*, *pccb*, and *lin28 *[24,25]; however, we found almost all of these *Alu *element-containing transcripts to be clearly classified as poly(A)+ in both cell lines. Therefore, transcripts with short encoded A stretches are not likely retained on oligo(dT) under our conditions.

Although it is hard without additional experimental support to predict how many of the classified transcripts truly contain two distinct transcripts, the information we provide here represents a comprehensive list of abundant transcripts that are potentially bimorphic.

### Incomplete transcripts do not significantly affect the population of bimorphic transcripts

**Figure 4:**
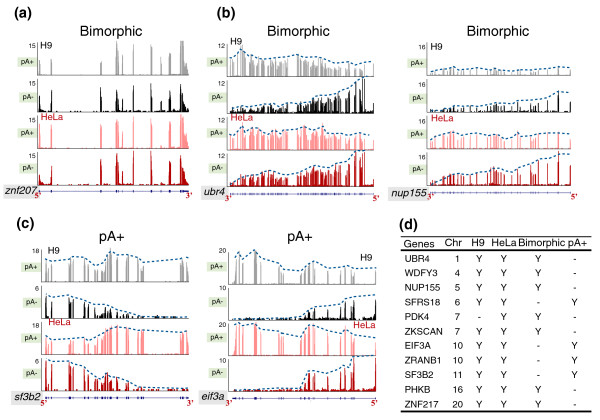
**Visualization of incomplete transcripts in the poly(A)- samples**. **(a) **A bimorphic example showing uniform coverage across the complete transcript. Note that the majority of the identified bimorphic transcripts are similar to this. **(b) **Examples of bimorphic transcripts with non-uniform coverage. Note that both *ubr 4 *(*retinoblastoma-associated factor 600*) and *nup155 *(*nucleoporin 155 kDa*) show similar normalized read densities in poly(A)+ and poly(A)- samples; however, both exhibit a gradual enrichment toward the 5' ends of the genes (blue dashed lines) in the poly(A)- samples. **(c) **Examples of non-poly(A)+ transcripts with non-uniform coverage. Note that both *sf3b2 *(*splicing factor 3b*, *subunit 2*) and *eif3a *(*eukaryotic translation initiation factor 3*) exhibit a gradual 5' end enrichment (blue dashed lines) in the poly(A)- samples, although both are more abundant in poly(A)+ samples (note the difference in the y-axis). **(d) **Examples of bimorphic and poly(A)+ transcripts with non-uniform coverage. See text for details.

Next, one could argue that the isolation of nascent or aborted transcripts or transcripts in the process of slow or partial 3' decay might also contribute to the pool of bimorphic transcripts. To address this we manually examined our sequencing data using the University of California, Santa Cruz (UCSC) genome browser. The vast majority of bimorphic transcripts we observed were like those shown in Figures 3c,d and 4a: alignments showed a similar pattern along the entire length of transcribed exons in both poly(A)+ and poly(A)- samples, indicating these transcripts contain the same sequences. However, we identified some RNAs that appeared to lack their annotated 3' ends. These were observed in the poly(A)- samples from both cell lines (Figure 4b,c; and Additional file 12). In the poly(A)- samples, these transcripts showed a pattern where few reads were aligned to the 3' ends but the read density increased toward the 5' ends of the genes (Figure 4b,c, compare black and red colors in poly(A)- (5' ends enriched) to grey and pink colors in poly(A)+). Thus, these mRNAs could be classified as bimorphic transcripts (Figures 3d (*gprc5a*) and 4b; Additional file 12a) or as poly(A)+ transcripts (Figure 4c; Additional file 12b-d), solely depending on the percentage of total transcripts that lack the 3' ends. For example, if half of the transcripts from a given gene show a 5'-end-enriched pattern, these transcripts would be classified as bimorphic transcripts (Figure 4b,d; Additional file 12a); however, if only a small fraction of the transcripts were of this type, they would be classified as poly(A)+ transcripts (Figure 4c,d; Additional file 12b-d). We found several dozen transcripts showing clear patterns of enriched 5' ends and a representative list of these is presented in Figure 4d. However, such molecules account for a small fraction of the total bimorphic RNAs. More interestingly, most of these events were detected in both H9 and HeLa cells (Figure 4d), suggesting that the 5' end enrichment could be an intrinsic nature for these transcripts, independent of the cell type. We note that such transcripts could arise from a variety of mechanisms, including slow 3' to 5' decay or incomplete nascent transcription, and at this time we cannot distinguish between these possibilities. We find no evidence that longer genes show this phenomenon more frequently than shorter genes, and the effects appear unrelated to transcript abundance.

### Characterization of poly(A)- transcripts

**Figure 5:**
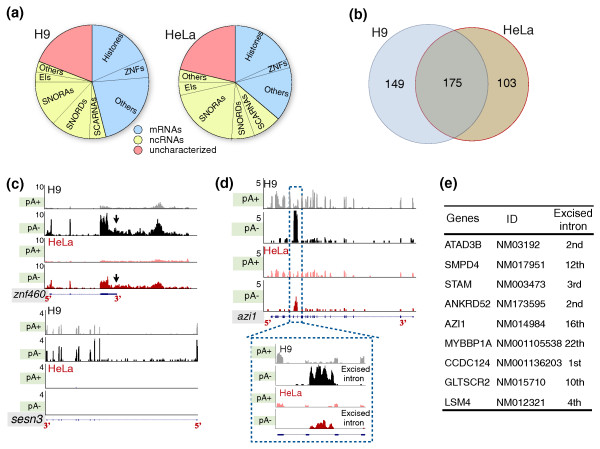
**Classification of poly(A)- transcripts**. **(a) **Classification of poly(A)- transcripts: EIs, excised introns; ZNF, zinc finger factor protein family. See text for details. **(b) **Overlapping analysis of the expression of poly(A)- transcripts in H9 and HeLa cells. **(c) **Example of a poly(A)- non-histone mRNA, *znf460 *and *sesn3*. The relative signals from either poly(A)+ or poly(A)- RNA preparations were normalized to those in the total RNA preparation in each cell line. Note that the signals from the poly(A)- samples are significantly enriched. Black arrows show the extended unannotated 3' UTR region of *znf460*. **(d) **An example of the excised 16th intron of the mRNA *azi1*. The blue box reveals the information in detail from this region. Note that the excised intron is abundant and can be detected only in the poly(A)- samples. **(e) **Examples of excised introns from different mRNAs, and the position of the excised intron in each mRNA is indicated.

Besides a significant amount of bimorphic transcripts, we also found 324 and 278 abundant long transcripts classified as poly(A)- in H9 and HeLa cells, respectively (Figure 1d). We note that this population may include both transcripts completely lacking poly(A) tails as well as those with very short tails. In addition to the known histone mRNAs, Cajal body related RNAs, and other known poly(A)- transcripts, we identified many uncharacterized transcripts and a group of mRNAs lacking a poly(A) tail, in which mRNAs for ZNF proteins were significantly overrepresented (Figure 5a). In contrast to the transcripts described above that have enriched coverage at their 5' ends in the poly(A)- sample only, the coverage across these transcripts is similarly uniform in the poly(A)+ and poly(A)- samples in both cell lines (Figure 5c, compare black to grey in H9 cell and red to pink in HeLa cells in both *zinc finger protein 460 *(*znf460*) and *sestrin 3 *(*sesn3*)). Interestingly, our deep sequencing data also revealed that some of these poly(A)- transcripts (and bimorphic transcripts as well) contain 3' UTRs that extend beyond the currently annotated ends of the genes (for a poly(A)- example, see Figure 5c, upper panel, *znf460*; for a bimorphic example, see Figure 3d, bottom right panel, *gprc5a*). The possibility thus exists that some of these transcripts may be detected in the poly(A)- fraction due to inefficient or alternative polyadenylation resulting in the production of RNAs either lacking poly(A) tails or containing short poly(A) tails. It is also possible that this is not a biological problem, but rather one of incomplete annotation.

### Stable excised introns are a new class of long non-coding RNAs

Interestingly, a number of stable excised introns were discovered by manually analyzing our data on the UCSC genome browser. These excised introns were observed in the poly(A)- RNA samples from both H9 and HeLa cells, and therefore could represent a new class of lncRNAs lacking poly(A) tails (Figure 5a,d,e; Additional file 13). Figure 5d shows one example of the excised 16th intron of the *azi1 *(*5-azacytidine induced 1*) mRNA (*EI-azi1*). *EI-azi1 *accumulates in both H9 and HeLa cells and is only detected in the poly(A)- RNA samples. Figure 5e and Additional file 13 offer a representative list of such highly abundant excised introns from a variety of intron regions in different mRNAs. These abundant, stable excised introns are of different lengths and most can be detected in both tested cell lines. It is well known that the vast majority of excised introns are rapidly degraded after debranching. We do not yet know whether these represent introns that are inefficiently debranched, or whether their accumulation results from specific *cis*-elements or the association with stabilizing proteins.

In addition to excised introns, we also observed the curious accumulation of several specific exons from internal regions of genes (Additional file 14). In the cases shown, one or two adjacent exons are extremely abundant in the poly(A)- RNA samples, while adjacent exon regions are not. Again, this occurs in samples from both cell lines. Although the mechanisms of formation of these RNAs are unknown, further studies will be focused on their biogenesis and whether these excised introns and exons have specific cellular locations or any specific biological functions.

### Specific expression of a group of histone genes in hESCs

**Figure 6:**
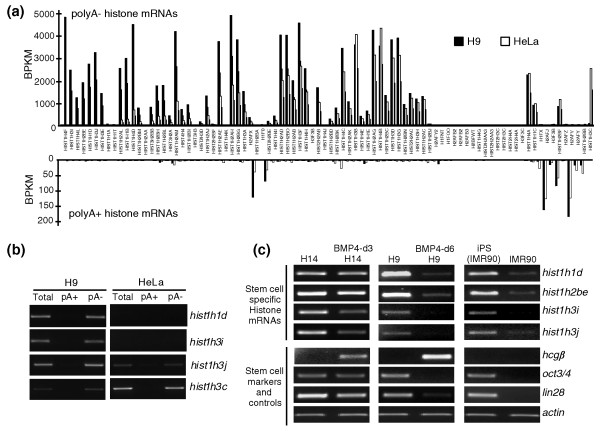
**Some histone genes are specifically expressed in hESCs and are preferentially associated with pluripotency**. **(a) **The relative expression (normalized read densities) of all histone genes in both H9 and HeLa cells. Note that a number of histone genes showed significantly higher expression in H9 cells compared to that in HeLa cells, while a few showed a HeLa cell-specific expression pattern. **(b) **Validation of cell-specific histone mRNA expression by semi-quantitative RT-PCR using RNAs prepared according to their 3' end status. **(c) **Pluripotency-associated histone gene expression. Total RNAs were collected from different cell lines or cells treated under differentiation or reprogramming conditions, and were then treated with DNaseI before being subjected to semi-quantitative RT-PCR analysis. Some histone mRNAs (*hist1h1d*, *hist1h2b*, *hist1h3i*, and *hist1h3j*) were found to be preferentially expressed in undifferentiated stem cells and in reprogrammed cells, but their expression rapidly decreased upon differentiation and was low prior to reprogramming. *hcgβ *is a marker for trophoblast differentiation; *oct3/4 *and *lin28 *are pluripotency markers; and *actin *was used as a loading control.

While we expected to observe histone mRNAs in the poly(A)- fractions, we were surprised to find different profiles of histone gene expression between HeLa cells and hESCs. Comparison of the relative expression of poly(A)- transcripts in H9 and HeLa cell lines revealed that approximately 60% are expressed in both cell lines (Figure 5b; Additional file 15); however, some poly(A)- histone transcripts we identified are specifically expressed in H9 cells (Figure 6a; Additional files 16 and 17).

The majority of histone genes are expressed as replication-dependent, poly(A)- transcripts. Interestingly, although most histone mRNAs are expressed in all somatic cells, different cell types have been found to express alternative histones [26-29]. More importantly, several recent observations have suggested that the state of chromatin in undifferentiated stem cells appears to be quite different from that of differentiated cells - these cells show a more diffuse and 'hyperdynamic' heterochromatin structure [30] and some histone modifications on the chromatin are likely to be bivalent [31]. Further, pluripotency may be coupled to a unique cell cycle program characterized by rapid proliferation and a truncated G1 phase [32-34]. As such, the cells devote more than half of the entire cell cycle to S phase and may lack a G1/S checkpoint. Since histone expression is mechanistically coupled to S-phase progression, it is perhaps not surprising to find distinct histone expression in pluripotent cells. Strikingly, however, we found that at least ten poly(A)- histone transcripts are preferentially expressed in H9 cells when compared to HeLa cells (fold change >10, *P *< 0.05), and one poly(A)- histone transcript is preferentially expressed in HeLa cells (Figure 6a,b; Additional files 16 and 17). In contrast, the expression levels of all poly(A)+ histone transcripts are comparable in both cell types (Figure 6a), although their expression levels are much lower than those of the poly(A)- histone transcripts, consistent with their roles in replication-independent expression [27,29].

While H9 cells express a number of histone genes that are poorly expressed in HeLa cells, it is important to note that, with the exception of two histone H1 variants (*hist1h1b *and *hist1h1d*), all of these genes express proteins that are identical or nearly identical to histones expressed from other loci (data not shown). This suggests that undifferentiated H9 cells may simply require a higher dosage of replication-dependent histone gene expression in order to maintain rapid growth and self-renewal properties. However, the expression of distinct histone H1 variants may be important for the maintenance of the unique chromatin status of these cells. In addition, since some of these replication-dependent histones are expressed from the same gene clusters (Additional file 17), it will be of interest to determine how specific histone gene transcription is regulated in the different cell lines.

Finally, we examined the expression of the hESC-specific histone transcripts described above during H9 and H14 cell differentiation. We treated hESCs with bone morphogenetic protein (BMP)4, which leads to trophoblast lineage differentiation [25,35,36] and found that the expression of these histone transcripts was significantly diminished upon differentiation (Figure 6c). For example, early (3 days) after BMP4 treatment of H14 cells the stem cell marker genes *oct3/4 *and *lin28 *were still expressed and a trophoblast maker gene *hcgβ *was just beginning to be expressed. However, at this time we already observed a significant reduction in the expression of *hist1h3i *and *hist1h3j *in these cells (Figure 6c, lanes 1 and 2). Prolonged (6 days) BMP4 treatment revealed that expression of all of the hESC-specific histone RNAs was reduced to almost undetectable levels in H9 cells (Figure 6c, lanes 3 and 4). We note, however, that 6 days after induction of differentiation of hESCs by BMP4 the cells grew slowly. Therefore, a complementary approach was taken to address the issue of a connection between specific histone expression and pluripotency. Consistent with a specific pattern of histone gene expression in pluripotent cells, we also observed a similar expression pattern of hESC-specific histone gene transcription upon reprogramming of human fibroblast IMR90 cells (Figure 6c, lanes 5 and 6). The hESC-specific histone mRNAs were expressed at extremely low levels in precursor human diploid IMR90 cells, while their expression significantly increased upon reprogramming to induced pluripotent stem (iPS) cells. Taken together, these observations suggest that a specific group of histone transcripts might serve as a novel group of sensitive pluripotency markers. As these histone transcripts are not abundantly expressed in other dividing cells such as HeLa cells and primary IMR90 cells, the possibility exists that these specific histones are functionally connected to the unique chromatin status of undifferentiated stem cells.

## Conclusions

We have used deep sequencing to explore the repertoire of both poly(A)+ and poly(A)- RNAs from two standard cell lines, HeLa cells and hESC H9 cells. This work provides a resource for not only the discovery but also for the study of many novel aspects of gene regulation. We found while the majority of the transcripts are poly(A)+, a significant portion of transcripts are either poly(A)- or bimorphic. Our sequencing data not only allow us to show that a number of mRNAs that are important for many important biological processes may contain short poly(A) tails (Figures 3 and 5), but also provide a useful tool to visualize some transcripts showing 5' or 3' end enrichment (Figure 4; Additional files 7 and 12). Furthermore, we also identified excised introns as a new class of stable non-polyadenylated lncRNAs (Figure 5d,e; Additional file 13). Finally, in addition to the identification of poly(A)- mRNAs and non-coding RNAs, we found that a specific subset of poly(A)- histone mRNAs are expressed in undifferentiated hESCs and are rapidly diminished upon differentiation (Figure 6). Further, these same histone genes are induced upon reprogramming of fibroblasts to iPS cells. In conclusion, we offer a rich source of data that allows a deeper exploration of the poly(A)- landscape of the eukaryotic transcriptome. This approach can also be applied to the analysis of the poly(A)- transcriptomes of other model organisms.

## Materials and methods

### Cell culture and differentiation

HeLa cells were cultured under standard conditions. hES H9, H14 cell lines and iPS cell lines were maintained on plates coated with Matrigel (BD Biosciences, Bedford, MA, USA) in either defined mTeSR medium (StemCell Technologies Inc., Vancouver, BC, Canada) or conditioned medium with irradiated mouse embryo fibroblasts supplemented with 4 ng/ml human basic fibroblast growth factor (Life Technologies, Inc., Grand Island, NY, USA) [25,35,36]. Passages 2 to 6 of IMR90 cells were used in this study. For trophoblast differentiation, hESCs were treated with 100 ng/ml BMP4 (R&D Systems, Minneapolis, MN, USA) in the presence of conditioned medium and basic fibroblast growth factor for the indicated days [25,35,36]. Human iPS (IMR90) cell lines were generated from IMR90 precursor cells and were verified at the UConn Stem Cell Core [37,38] and confirmed positive for Tra-1-81, Tra-1-60, SSEA-3 and SSEA-4 by immunofluorescence and teratoma formation [38]. Pluripotent cell cultures were regularly evaluated for Oct3/4 expression every 3 to 4 weeks and cells were passaged every 6 to 7 days.

### Poly(A)+ and poly(A)- RNA separation

Total RNAs were prepared using Trizol Reagent (Life Technologies, Carlsbad, CA, USA). After treatment with DNase I (DNA-free kit; Ambion, Austin, TX, USA), total RNAs were incubated with oligo(dT) magnetic beads to isolate either poly(A)+ RNAs, which were bound to beads, or poly(A)- RNAs, which were present in the flowthrough after incubation. Oligo(dT) magnetic bead selection was performed three times to ensure pure poly(A)+ or poly(A)- populations. The poly(A)- RNA population was further processed with the RiboMinus kit (Human/Mouse Module, Invitrogen, Carlsbad, CA, USA) to deplete most of the abundant ribosomal RNAs (Figure 1).

### Sequencing

All RNA-Seq libraries were prepared using the Illumina mRNA-Seq Sample Prep Kits (P/N 1004814) according to the manufacturer's instructions. Briefly, poly(A)- or poly(A)+ RNAs were fragmented using divalent cations at elevated temperature, reverse transcribed with random hexamers to obtain double-stranded cDNA fragments, which were end-repaired and 5' end phosphorylated. After adding 'A' bases to the 3' ends, Illumina adaptor oligonucleotides were ligated to the cDNA fragments and approximately 300-bp fragments were isolated from an agarose gel, followed by PCR amplification and gel purification. The cDNA libraries were then individually loaded onto flowcells for cluster generation (version 2) after quantification with Nanodrop, and sequenced on an Illumina Genome Analyzer II*x *using a single-read protocol of 75 cycles with v3 chemistry. All sequence files can be accessed from the NCBI Sequence Read Archive by Gene Expression Omnibus accession number [GEO:GSE24399].

### Alignments

The poly(A)+ or poly(A)- sequence reads were uniquely aligned to the human hg19 genome and splice junction index by using Bowtie [18,19], allowing up to two mismatches. Wiggle track files were generated from bowtie output files by a custom bowtie2wiggle script and correlations among different samples were calculated with MATLAB. Replicate lanes were then concatenated and viewed on the UCSC genome browser. The normalized read density of each gene was set for comparison on the UCSC genome browser with a normalized read density value. Since the size of the wiggle tracks of the concatenated poly(A)+ samples for both cell lines exceeded the limit of the UCSC genome browser tracks for uploading, we compared one poly(A)+ sequencing dataset from each cell line to the concatenated poly(A)- sample of that cell for visualization purposes. Thus, while all data were used for analysis, a single sequencing round for poly(A)+ RNA was used for visualization purposes but concatenated data were used for poly(A)- visualizations. Normalized gene expression levels were determined in units of BPKM for all 27,297 annotated genes (hg19, 2009, UCSC) using wig_integrator.pl (Additional files 3 and 4). BPKM is the simple sum (integral) of base coverage over the limits defined by a given feature (exon, transcript, gene) from the annotated genome and represents the integral of the wiggle track over feature interval limits, which is then normalized by total aligned bases and the length of the feature.

### Classification

Genes were classified into different subgroups according to their 3' end structures using several parameters, including BPKM values for expression level, fold changes of poly(A)- reads verse poly(A)+ reads, and *P*-value of fold change determined by Wald test analysis with a custom perl script.

#### Poly(A)- predominant subgroup

For each gene in this subgroup, the BPKM value from a poly(A)- sample must be ≥1, the fold change of the BPKM value of poly(A)- versus the BPKM value of poly(A)+ must be ≥2, and the *P*-value of fold change must be <0.05 (Wald score >1.96).

#### Poly(A)+ predominant subgroup

For each gene in this subgroup, the BPKM value from the poly(A)+ sample must be ≥1, the fold change of the BPKM value of poly(A)- versus the BPKM value of poly(A)+ must be ≤0.5, and the *P*-value of fold change must be <0.05 (Wald score <-1.96).

#### Bimorphic subgroup

For each gene in this subgroup, the BPKM value from the poly(A)+ sample or poly(A)- sample must be ≥1, the fold change of the BPKM value of poly(A)- versus the BPKM value of poly(A)+ must be between 0.5 and 2, and the *P*-value of the fold change must be <0.05 (Wald score >1.96 or <-1.96).

#### Subgroup with low expression and/or low significant changes

For each gene in this subgroup, the BPKM value is <1, and/or the *P*-value of fold change is >0.05 (-1.96 < Wald score < 1.96). This group also included genes for which there was no unique read aligned under the conditions used in this study. This group was not analyzed further in this study.

### Validation

RT-PCR and/or qRT-PCR were preformed from independent poly(A)- and poly(A)+ enriched samples from different cell lines for validation. Isolated RNA samples were resuspended in the same amount of DEPC-H_2_O and 1 μg of each sample was reverse transcribed to cDNAs using SuperScript II (Invitrogen) and random hexamers. In addition, cDNA from the same amount of unfractionated (total) RNA was also transcribed as a control. To be consistent, all semi-quantitative PCRs were amplified with either 26 or 28 cycles (depends on the relative abundance of specific transcripts in the transcriptome) to visualize the differences from different fractionations. For the real time PCR, the relative abundance of each tested transcript in either poly(A)- or poly(A)+ enriched samples was normalized to total RNA. We mathematically assumed the total RNA equals the poly(A)+ RNA plus poly(A)- RNA; therefore, if the signal in poly(A)- RNA was two-fold that in poly(A)+, it would count for two-thirds of the signal from the total RNA. Primer sets are listed in Additional file 18.

## Abbreviations

BMP: bone morphogenetic protein; BPKM: bases per kilobase of gene model per million mapped bases; hESC: human embryonic stem cell; iPS: induced pluripotent stem; lncRNAs: long non-coding RNAs; poly(A)- RNAs: non-polyadenylated RNAs; poly(A)+ RNAs: polyadenylated RNAs; qPCR: quantitative PCR; rRNA: ribosomal RNA; snRNA: small nuclear RNA; UCSC: University of California, Santa Cruz;UTR: untranslated region; ZNF: zinc finger proteins.

## Authors' contributions

LY and LLC designed the experiments, performed the experiments, and performed the statistical analysis with perl scripts written by MOD; LY, GGC, and LLC collected the data; LY, BRG, GGC and LLC wrote the paper.

## Supplementary Material

Additional file 1 **Correlation among poly(A)+ and poly(A)- samples in H9 and HeLa cells**. The scale for all plots is log_10_. 1-3, the sequencing triplicates of sample poly(A)- transcripts in H9 cells; 4-6, the sequencing triplicates of sample poly(A)+ transcripts in H9 cells; 7-9, the sequencing triplicates of sample poly(A)- transcripts in HeLa cells; 10-12, the sequencing triplicates of sample poly(A)+ transcripts in HeLa cells. Note the linear correlation among triplicates of individual samples is greater than 98%. Click here for file

Additional file 2 **Read counts and coverage per concatenated sample**. Click here for file

Additional file 3 **Full list of genes and deep sequencing results from HeLa cells**. Reads from poly(A)- (#1) and poly(A)+ (#2) samples are normalized for BPKM. Genes are sorted by their Wald scores (WaldStat). Click here for file

Additional file 4 **Full list of genes and deep sequencing results from H9 cells**. Reads from poly(A)- (#1) and poly(A)+ (#2) samples are normalized for BPKM. Genes are sorted by their Wald scores (WaldStat). Click here for file

Additional file 5 **List of poly(A)- genes from HeLa cells**. Criteria for inclusion in this list are described in the text. Normalized reads from poly(A)- and poly(A)+ samples. Genes are sorted by their Wald scores (WaldStat). Click here for file

Additional file 6 **List of poly(A)- genes from H9 cells**. Criteria for inclusion in this list are described in the text. Reads from poly(A)- and poly(A)+ samples are normalized. Genes are sorted by the Wald score (WaldStat). Click here for file

Additional file 7 ***Malat1 *and *neat1 *are examples of poly(A)- and bimorphic long non-coding RNAs**. **(a) ***Malat1 *exists in both poly(A)+ and poly(A)- isoforms and the poly(A)- isoform is more abundant than the poly(A)+ isoform. Fewer reads from the 5' end in the poly(A)- fraction are aligned to the genome in both cell lines. *Malat1 *is also more abundantly expressed in HeLa cells than in H9 cells. **(b) **Semi-quantitative RT-PCR with two sets of primers confirmed that *malat1 *is more abundant in poly(A)- samples. **(c) **Deep sequencing reveals that both isoforms of *neat1 *are undetectable in H9 cells. In HeLa cells, while the shorter isoform of *neat1 *is entirely poly(A)+ (pink color), the longer isoform is more enriched in the poly(A)- fraction (red), also seen in **(d) **for its relative abundance with the same y-axis in HeLa cells. Click here for file

Additional file 8 **List of bimorphic genes from HeLa cells**. Criteria for inclusion in this list are described in the text. Reads from poly(A)- and poly(A)+ samples are normalized. Genes are sorted by their Wald scores (WaldStat). Click here for file

Additional file 9 **List of bimorphic genes from H9 cells**. Criteria for inclusion in this list are described in the text. Reads from poly(A)- and poly(A)+ samples are normalized. Genes are sorted by their Wald scores (WaldStat). Click here for file

Additional file 10 **List of bimorphic genes that overlap in both H9 and HeLa cells**. Click here for file

Additional file 11 **The 3' end of the shorter isoform of *h2afx *contains the canonical consensus sequence within the 3' UTR of non-polyadenylated histone genes**. **(a) **MEME analysis (Multiple Em for Motif Elicitation) [22,23] revealed the consensus sequence within the 3' UTR regions of histone genes for poly(A)- RNAs. **(b) **MFold analysis (version 3.5, M Zuker, Rensselaer Polytechnic Institute) predicted the stem-loop structure within the 3' UTR of histone genes for poly(A)- RNAs. Click here for file

Additional file 12 **Visualization of transcripts exhibiting 3**' **decay in poly(A)- samples**. **(a) **Examples of 3' decay in the bimorphic group. *pdk 4 *(*pyruvate dehydrogenase kinase*, *isozyme 4*) is expressed in HeLa cells only and shows similar normalized read densities in poly(A)+ and poly(A)- samples; however, it exhibits a gradual decay pattern (blue dashed lines) from 3' to 5' in the poly(A)- sample in HeLa cells. **(b-d) **Examples of 3' decay in the poly(A)+ group. Note that *sfrs18 *(*splicing factor*, *arginine/serine-rich 18*), *sf3b2 *(*splicing factor 3b*, *subunit 2*) and *eif3a *(*eukaryotic translation initiation factor 3*) all exhibit a gradual decay pattern (blue dashed lines) from the 3' to 5' ends in poly(A)- samples in both H9 and HeLa cells, although both are more abundant in poly(A)+ samples. See text for details. Click here for file

Additional file 13 **Excised introns**. **(a) **The 12th intron of *smpd4 *(*sphingomyelin phosphodiesterase 4*) mRNA is an excised intron (blue dashed box) and accumulates in the poly(A)- sample in H9 cells. **(b) **The second intron of *atad3b *(*ATPase family*, *AAA domain containing 3B*) mRNA accumulates in the poly(A)- samples in both H9 and HeLa cells (blue dashed box). Click here for file

Additional file 14 **Poly(A)- exons**. **(a) **The second and third exons of *camsap1 *(*calmodulin regulated spectrin-associated protein 1*) mRNA accumulate in poly(A)- samples from both H9 and HeLa cells (blue dashed box). **(b) **Examples of poly(A)- exons in mRNAs, and the positions of the exons in mRNAs are indicated. Click here for file

Additional file 15 **List of poly(A)- genes that overlap in both H9 and HeLa cells**. Click here for file

Additional file 16 **Histone mRNAs preferentially expressed in H9 cells (*hist1h1d*, *hist1h3i*, and *hist1h3j*; black) or HeLa cells (*hist1h3c*; red)**. Only unique alignments were allowed. See Figure 6c for the relative transcription of these genes upon differentiation or reprogramming. Click here for file

Additional file 17 **A schematic view of histone gene cluster 1 on chromosome 6 comparing the expression of histone genes in H9 and HeLa cells**. While most histone genes (*hist1h2bl*, *hist1h2ai*, *hist1h3h*, *hist1h2ai*, *hist1h2bm*, *hist1h2ak*, *hist1h2bn*, *hist1h2am *and *hist1h2bo*) are expressed in both cell lines, some histone genes (*hist1h2al*, *hist1h1b*, *hist1h3i*, *hist1h4l *and *hist1h3j*) are expressed at significantly higher levels in H9 cells. Click here for file

Additional file 18 **Gene-specific primers used for RT-PCR and qPCR validation**. Click here for file
