# Supplementary material for: Genomewide characterization of non-polyadenylated RNAs
Source: Genome Biol. 2011 Feb 16;12(2):R16. doi: 10.1186/gb-2011-12-2-r16 (PMC3188798; doi:10.1186/gb-2011-12-2-r16)
Supplement: Additional file 2 — Read counts and coverage per concatenated sample. [file gb-2011-12-2-r16-S2.DOC]

**Additionsl**

**Additional file 2**

Alignments of RNA-Seq reads to the H.Sapiens genome (GRCh37/hg19) and splice junction index

H9 poly(A)-

H9 poly(A)+

HeLa poly(A)-

HeLa poly(A)+

37,193,134

39,346,501

45,643,430

53,833,115

4,995,064

22,955,544

6,196,492

33,383,663

13.4%

58.3%

13.6%

62.0%

Coverage%

Reads

Unique %

Unique

3.3%

5.5%

3.8%

6.8%
