# Supplementary figures and images for: Genomewide characterization of non-polyadenylated RNAs
Source: Genome Biol. 2011 Feb 16;12(2):R16. doi: 10.1186/gb-2011-12-2-r16 (PMC3188798; doi:10.1186/gb-2011-12-2-r16)

**A**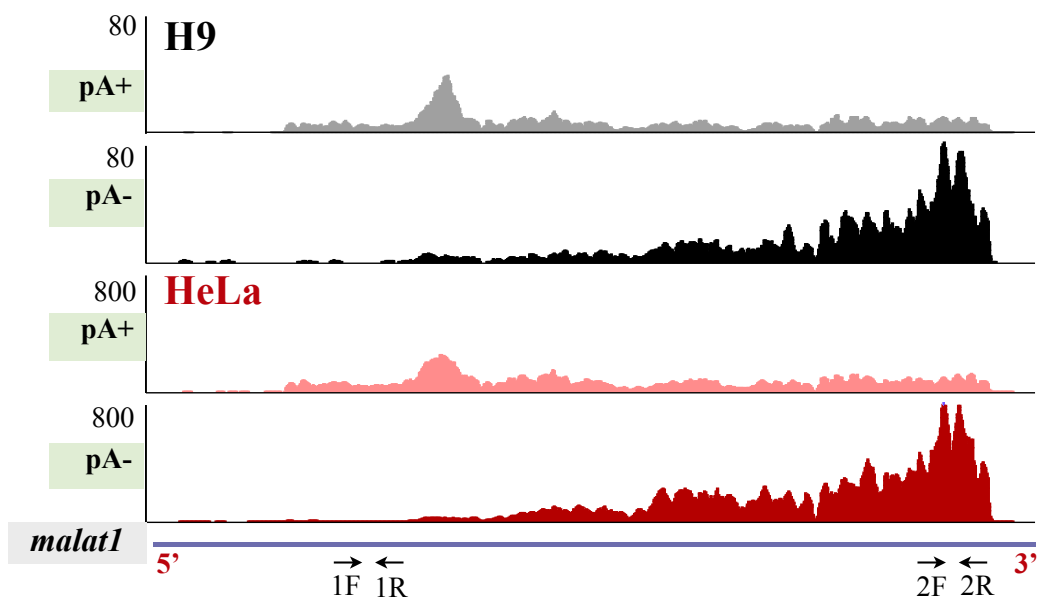**B**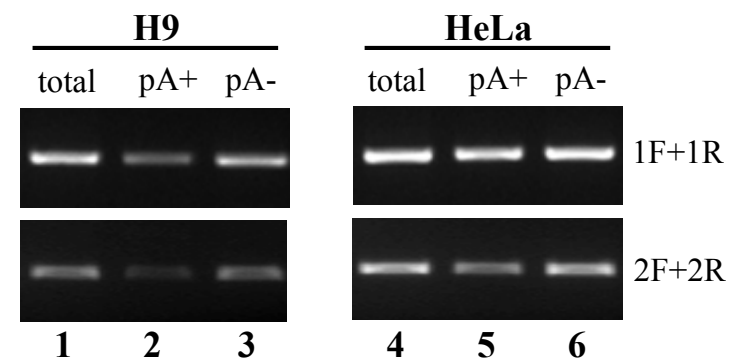**C**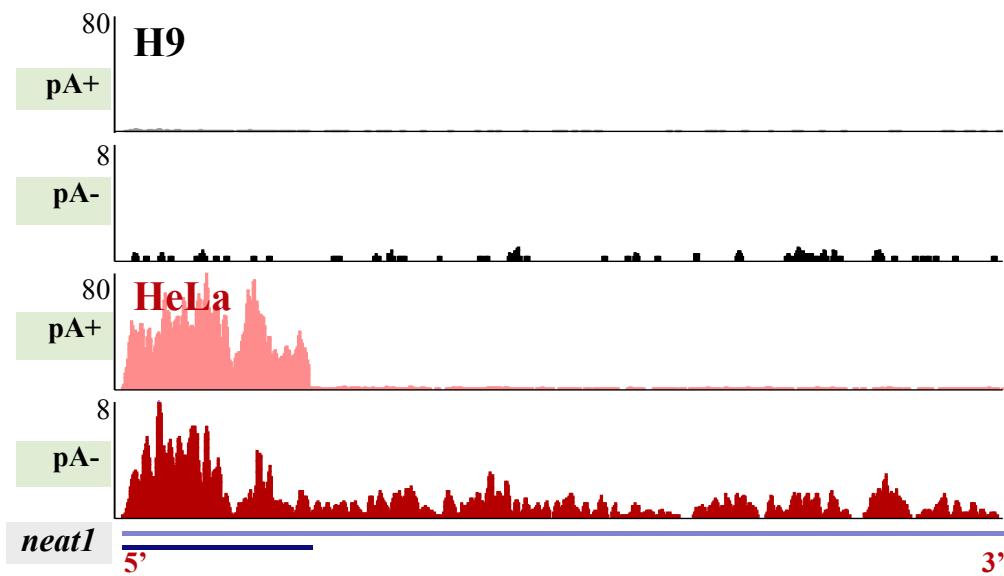**D**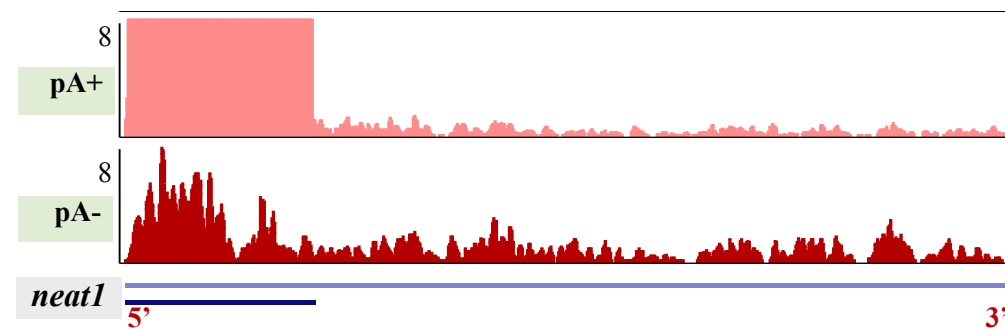

Supplement: Additional file 7 — Malat1 and neat1 are examples of poly(A)- and bimorphic long non-coding RNAs. (a) Malat1 exists in both poly(A)+ and poly(A)- isoforms and the poly(A)- isoform is more abundant than the poly(A)+ isoform. Fewer reads from the 5' end in the poly(A)- fraction are aligned to the genome in both cell lines. Malat1 is also more abundantly expressed in HeLa cells than in H9 cells. (b) Semi-quantitative RT-PCR with two sets of primers confirmed that malat1 is more abundant in poly(A)- samples. (c) Deep sequencing reveals that both isoforms of neat1 are undetectable in H9 cells. In HeLa cells, while the shorter isoform of neat1 is entirely poly(A)+ (pink color), the longer isoform is more enriched in the poly(A)- fraction (red), also seen in (d) for its relative abundance with the same y-axis in HeLa cells. [file gb-2011-12-2-r16-S7.PDF]

**A**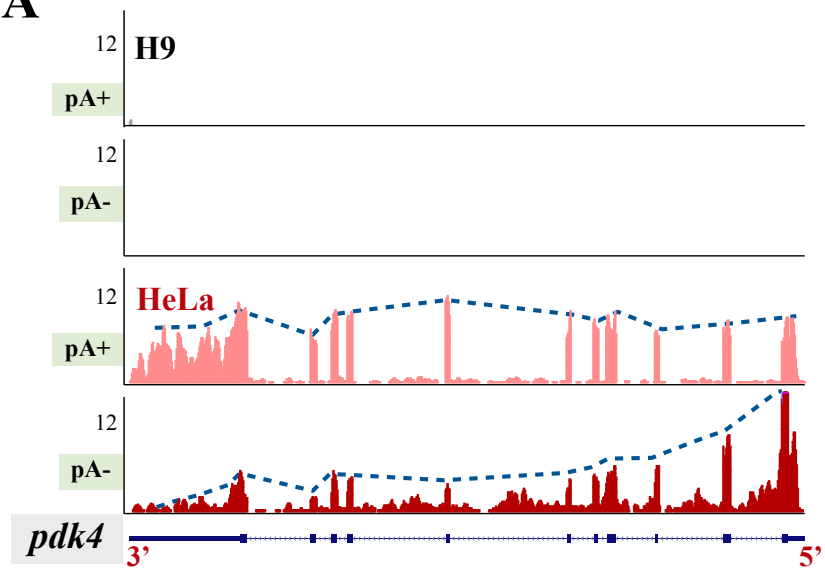**B**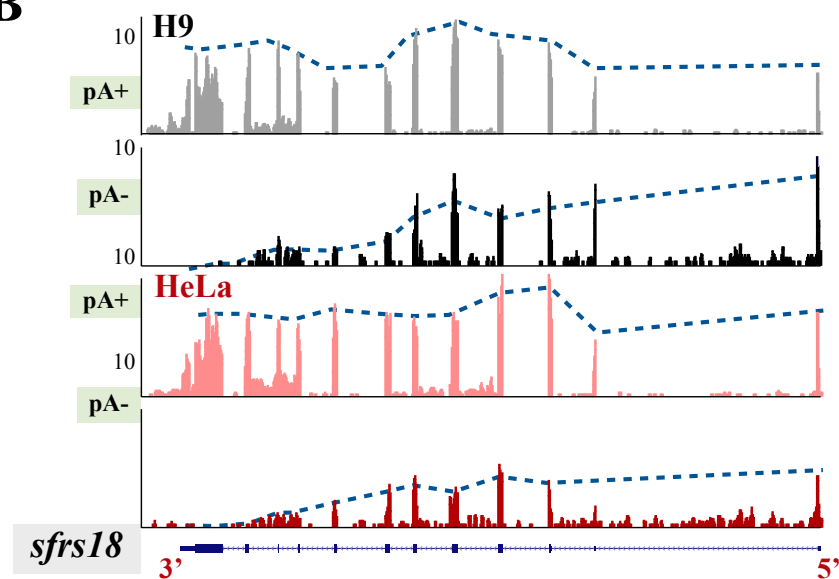**C**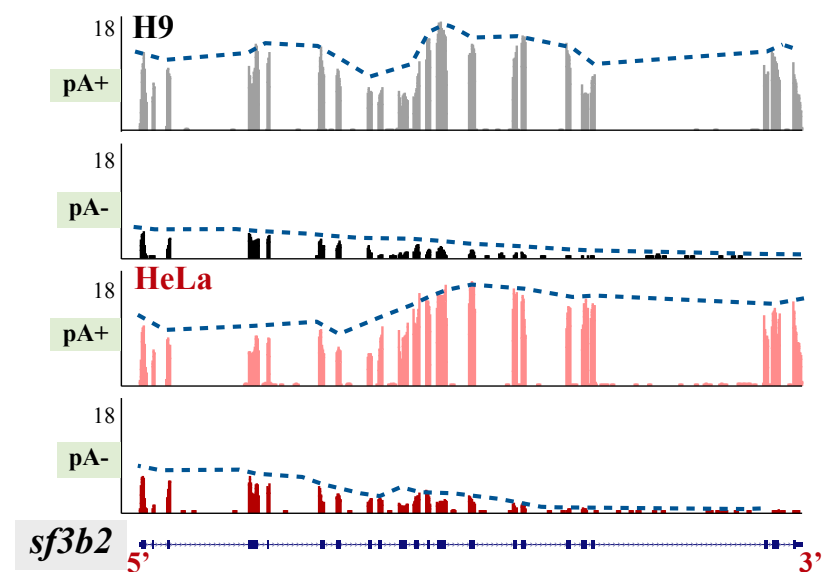**D**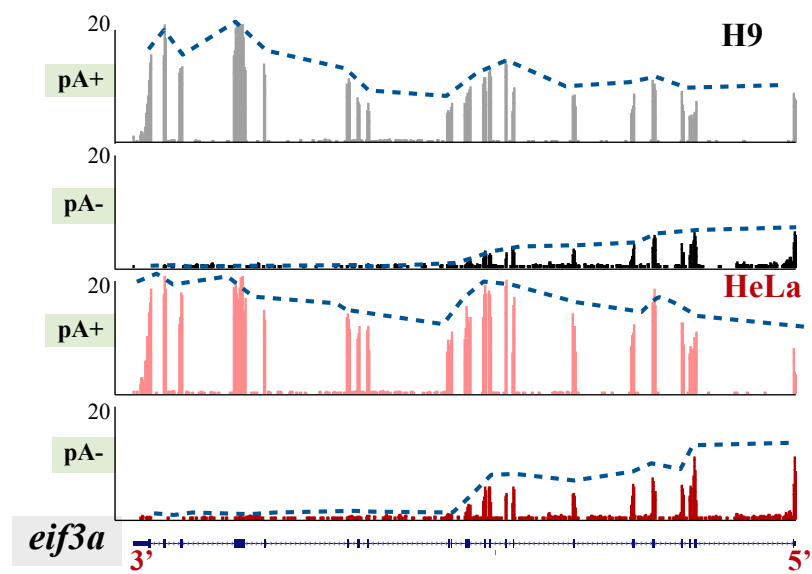

Supplement: Additional file 12 — Visualization of transcripts exhibiting 3' decay in poly(A)- samples. (a) Examples of 3' decay in the bimorphic group. pdk 4 (pyruvate dehydrogenase kinase, isozyme 4) is expressed in HeLa cells only and shows similar normalized read densities in poly(A)+ and poly(A)- samples; however, it exhibits a gradual decay pattern (blue dashed lines) from 3' to 5' in the poly(A)- sample in HeLa cells. (b-d) Examples of 3' decay in the poly(A)+ group. Note that sfrs18 (splicing factor, arginine/serine-rich 18), sf3b2 (splicing factor 3b, subunit 2) and eif3a (eukaryotic translation initiation factor 3) all exhibit a gradual decay pattern (blue dashed lines) from the 3' to 5' ends in poly(A)- samples in both H9 and HeLa cells, although both are more abundant in poly(A)+ samples. See text for details. [file gb-2011-12-2-r16-S12.PDF]

**A**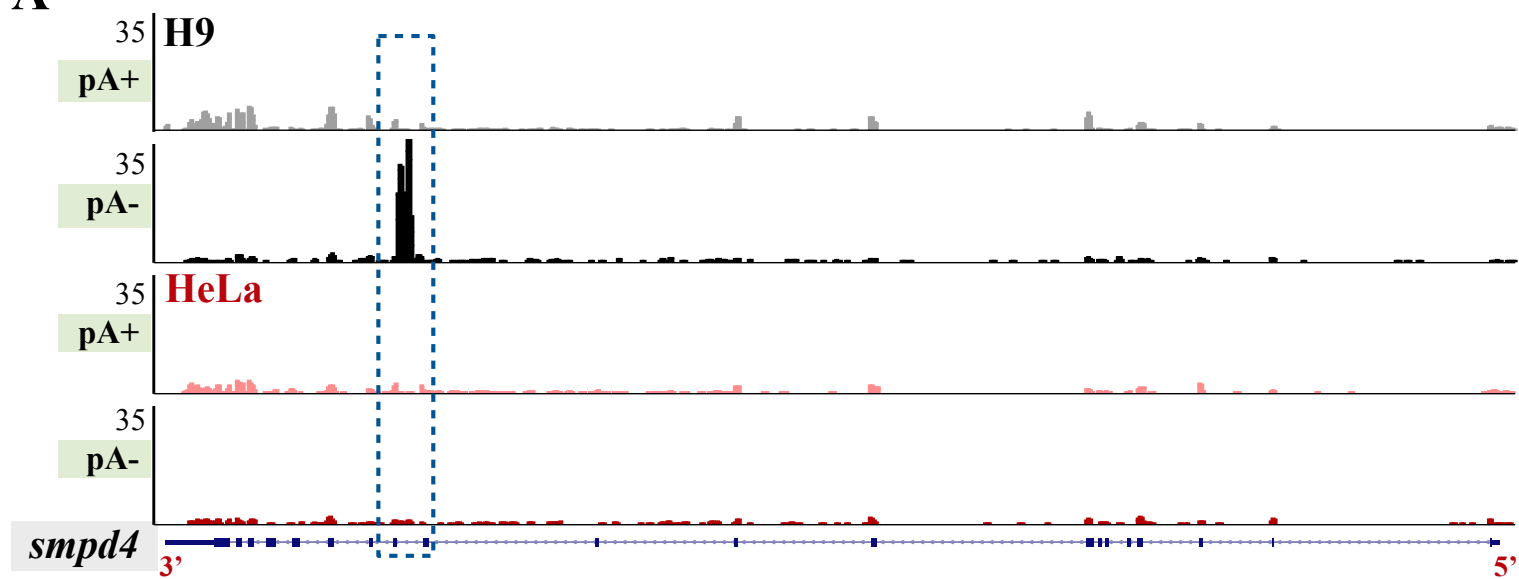**B**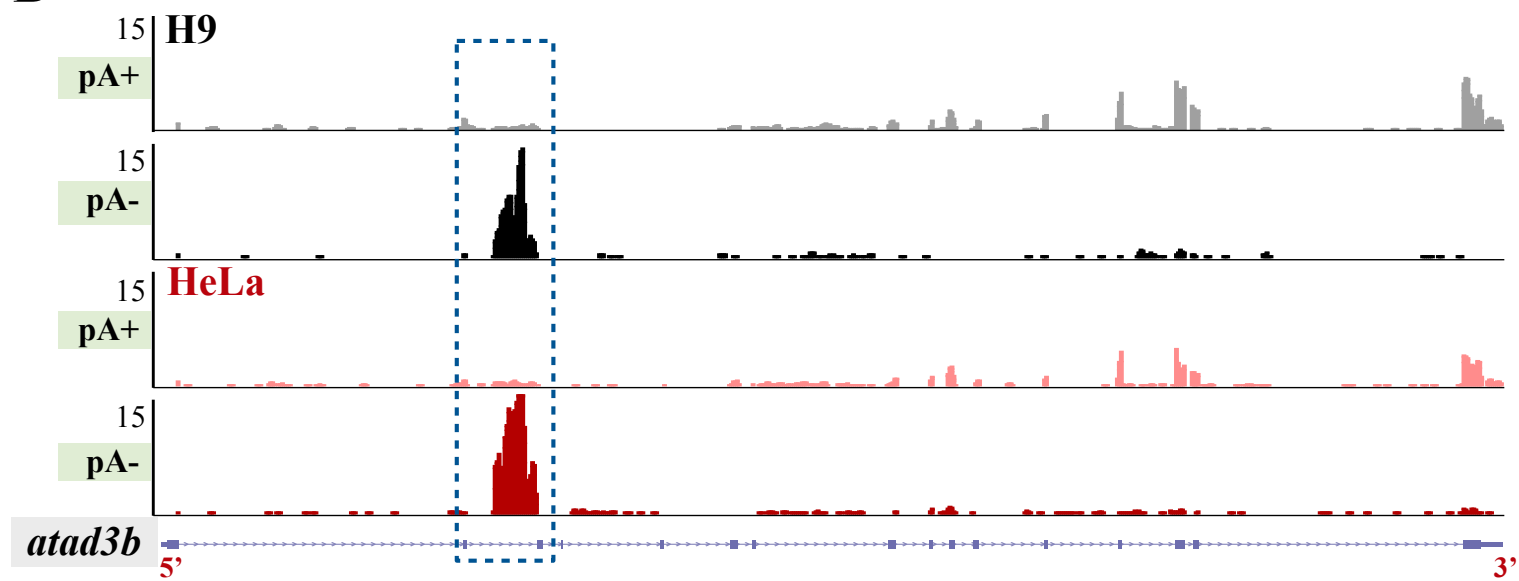

Supplement: Additional file 13 — Excised introns. (a) The 12th intron of smpd4 (sphingomyelin phosphodiesterase 4) mRNA is an excised intron (blue dashed box) and accumulates in the poly(A)- sample in H9 cells. (b) The second intron of atad3b (ATPase family, AAA domain containing 3B) mRNA accumulates in the poly(A)- samples in both H9 and HeLa cells (blue dashed box). [file gb-2011-12-2-r16-S13.PDF]

**A**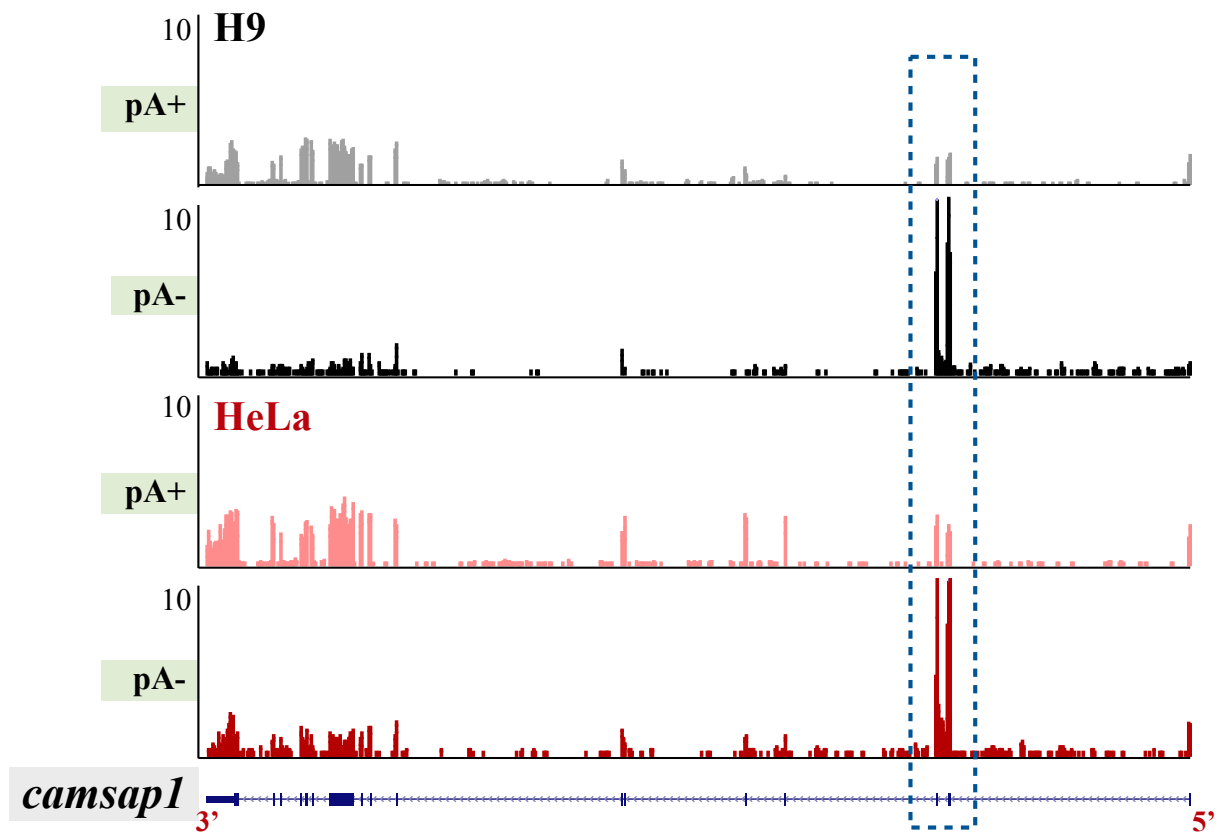**B**

| Genes    | Chr | Poly(A)- exons | H9 | HeLa |
|----------|-----|----------------|----|------|
| CAMSAP1  | 9   | 2nd and 3rd    | Y  | Y    |
| ARHGAP12 | 10  | 3rd            | Y  | Y    |
| PHC1     | 12  | 3rd and 4th    | Y  | -    |
| NFATC3   | 16  | 2nd and 3rd    | Y  | Y    |

Supplement: Additional file 14 — Poly(A)- exons. (a) The second and third exons of camsap1 (calmodulin regulated spectrin-associated protein 1) mRNA accumulate in poly(A)- samples from both H9 and HeLa cells (blue dashed box). (b) Examples of poly(A)- exons in mRNAs, and the positions of the exons in mRNAs are indicated. [file gb-2011-12-2-r16-S14.PDF]

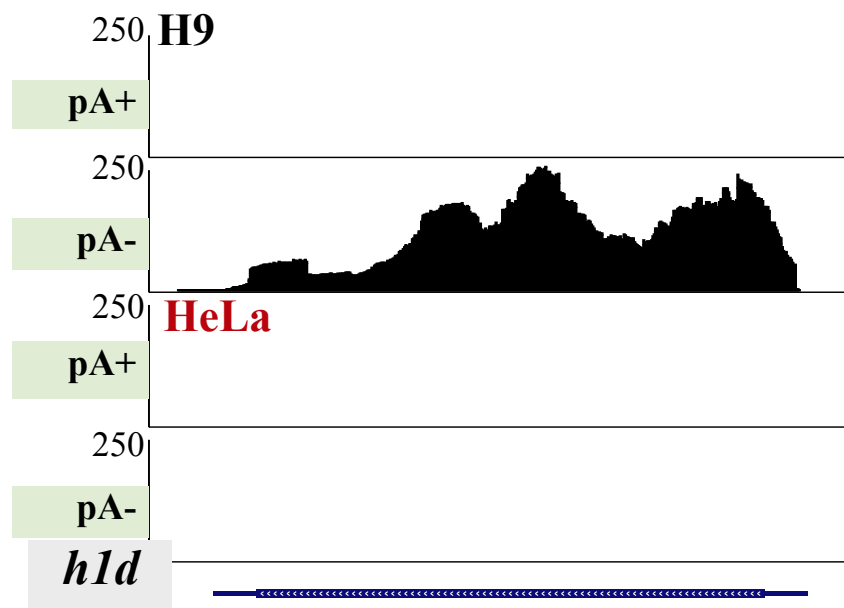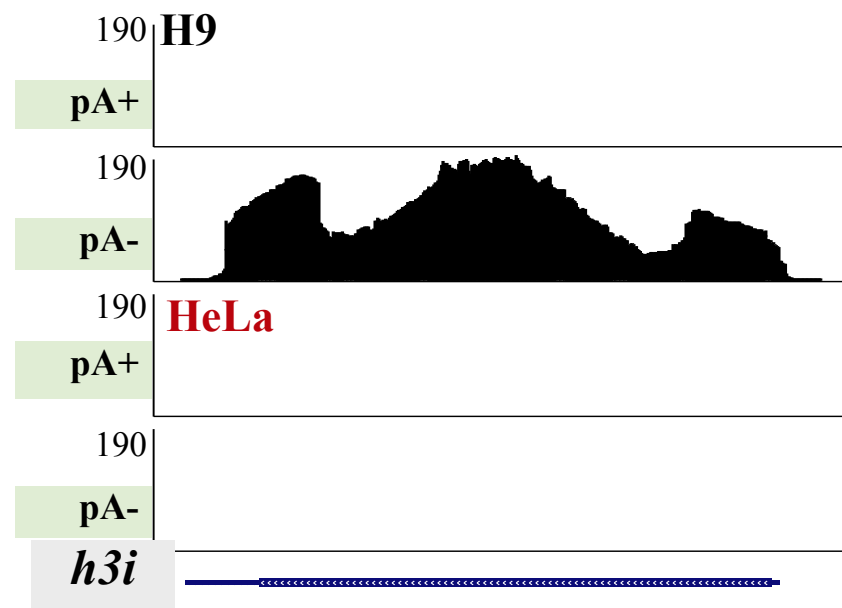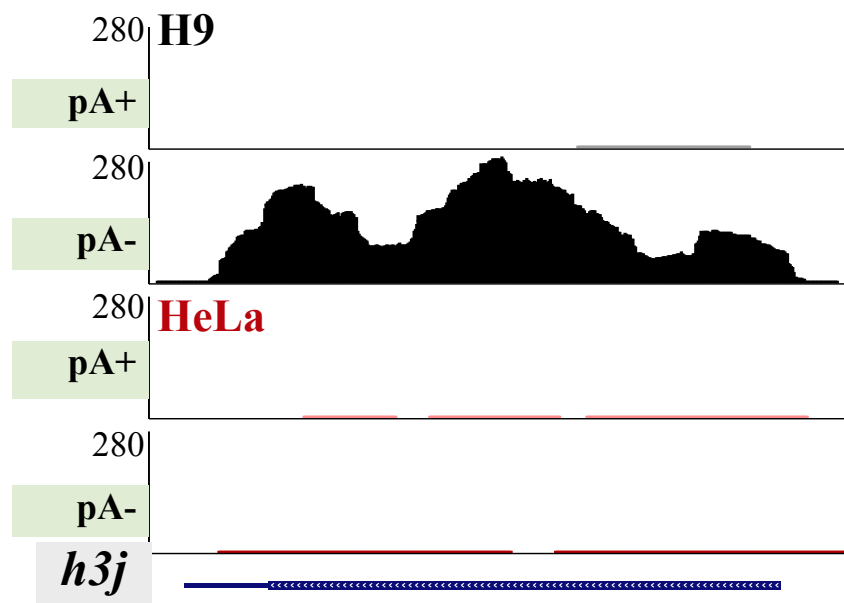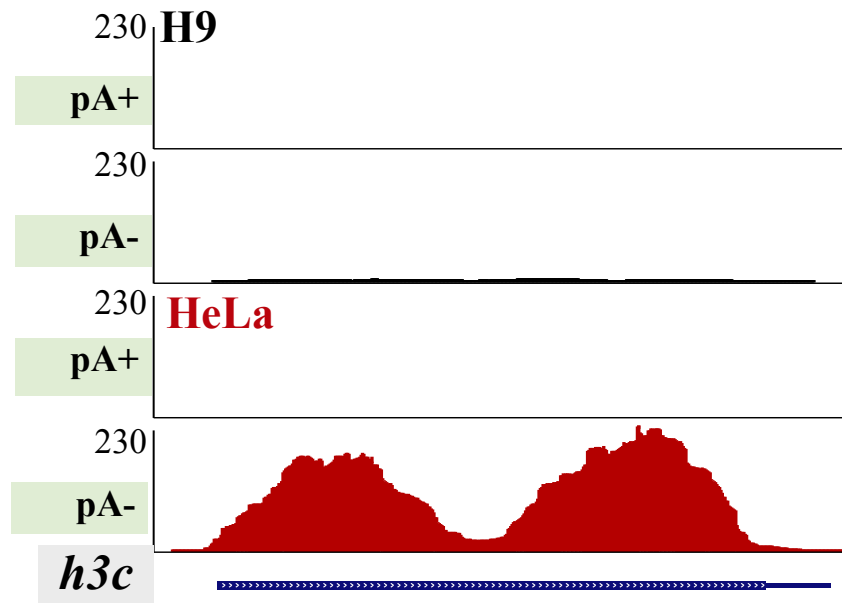

Supplement: Additional file 16 — Histone mRNAs preferentially expressed in H9 cells (hist1h1d, hist1h3i, and hist1h3j; black) or HeLa cells (hist1h3c; red). Only unique alignments were allowed. See Figure 6c for the relative transcription of these genes upon differentiation or reprogramming. [file gb-2011-12-2-r16-S16.PDF]

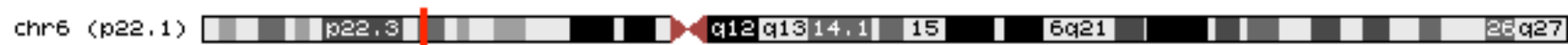

20 kb

**H9**

pA+

pA-

**HeLa**

pA+

pA-

*h2bl*  
*h2ai*  
*h3h*  
*h2aj*  
*h2bm*

*h4j*

*h4k*

*h2ak*  
*h2bn*

*h2al*  
*h1b*  
*h3i*  
*h4l*

*h3j*  
*h2am*  
*h2bo*

Supplement: Additional file 17 — A schematic view of histone gene cluster 1 on chromosome 6 comparing the expression of histone genes in H9 and HeLa cells. While most histone genes (hist1h2bl, hist1h2ai, hist1h3h, hist1h2ai, hist1h2bm, hist1h2ak, hist1h2bn, hist1h2am and hist1h2bo) are expressed in both cell lines, some histone genes (hist1h2al, hist1h1b, hist1h3i, hist1h4l and hist1h3j) are expressed at significantly higher levels in H9 cells. [file gb-2011-12-2-r16-S17.PDF]
