# Supplementary material for: Genomewide characterization of non-polyadenylated RNAs
Source: Genome Biol. 2011 Feb 16;12(2):R16. doi: 10.1186/gb-2011-12-2-r16 (PMC3188798; doi:10.1186/gb-2011-12-2-r16)

A

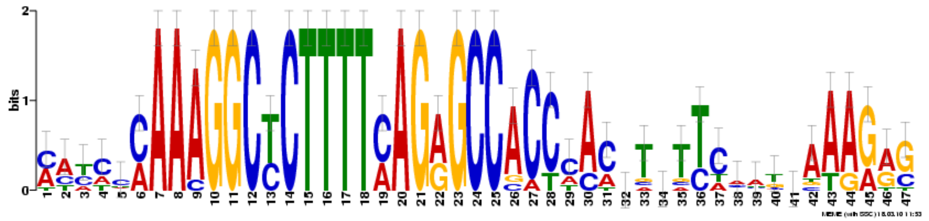

NAME	STRAND	START	P-VALUE	SITES
HIST1H2BF	+	22	6.24e-21	CTTCATACCC AATC CCAAAGGCTCTTTTAAGAGCCACCCA CTTTTTCAGCTATAGAG TTGTAATTAC
HIST2H2AC	+	29	1.01e-19	CATACTCATT CACC CCAAAGGCTCTTTTAAGAGCCACCAA AGTGTCAAATGAAGGGC TGATCACGAA
HIST1H2AD	+	40	1.26e-19	AGGAAGCAGT CTTA ACAAAGGCTCTTTTCAGAGCCACCCA TGTATTCCTTAAAAGGG CTCACATTTT
HIST1H4H	+	35	1.40e-19	ATTTTCATTT TCAA CCAAAGGCCCTTTTCAGGGCCGCCCA CTTTTTTCATAAAAGAG CAGACATCTT
HIST1H3D	+	29	5.93e-19	GTGCTAACCA AAAC CCAAAGGCTCTTTTCAGAGCCAACCA CCTTTTCTATAAAAGTT GCTGTTTACT
HIST1H3C	+	21	3.67e-18	GTTTCTTCCT CATT GAAAAGGCTCTTTTCAGAGCCACTCA CAATTTCACTTAAAAAC AGTTGTAACC
H2AFX	+	43	4.34e-18	CTCCCCATGC CACC ACAAAGGCCCTTTTAAGGGCCACCAC CGCCCTCATGGAAAGAG CTGAGCCGCT
HIST1H2BK	+	30	1.13e-17	CTTAAAGCCC AACC CCAAAGGCTCTTTTAAGAGCCACTTA AATTATCGATATTAGAG CTGTAAACAC
HIST4H4	+	27	1.53e-17	TTCTCTACAG ACTC CAAAAGGCCCTTTTCAGGGCCCCCAA ACTGTCACAGAAAGAGC TGTTAACACT
HIST1H1D	+	29	1.15e-16	CCCTTTGAAA ATTT TAAACGGCTCTTTTCAGAGCCACCCA CAGGTCTCAGTCAAAAG AGCTGAAGCT
HIST1H4F	+	34	1.22e-16	TAACAGCTCA CCCA TAAAAGGCCCTTTTCAGGGCCACCTC CTTCGTCACACGAAGGG CTGTAACTGA

B

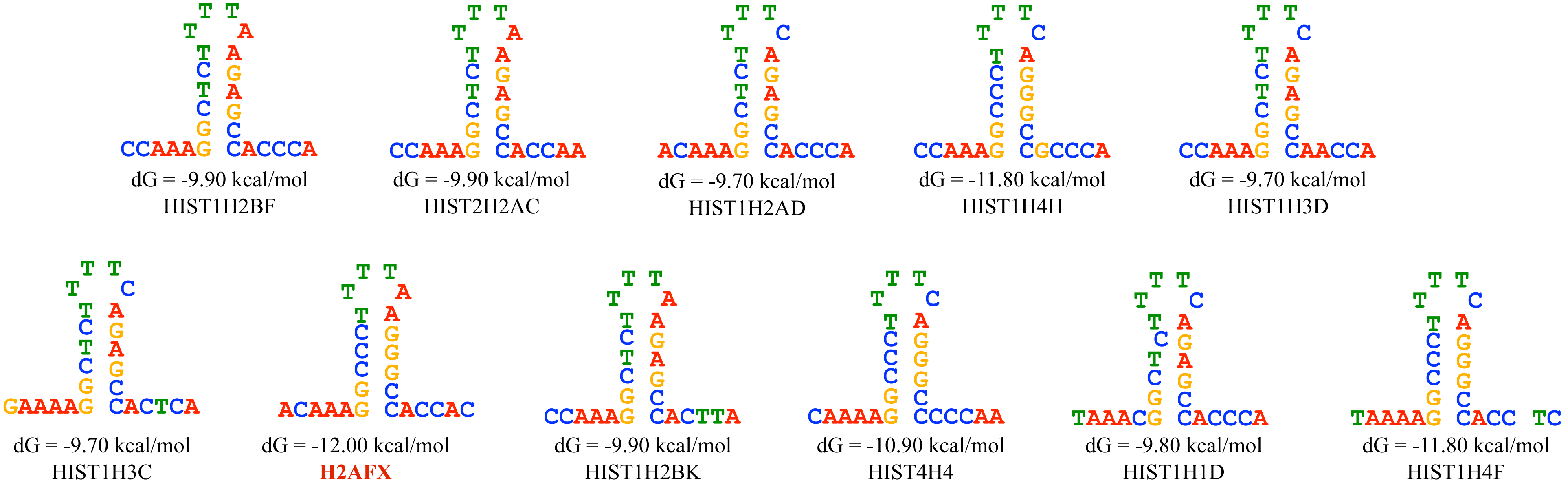

Supplement: Additional file 11 — The 3' end of the shorter isoform of h2afx contains the canonical consensus sequence within the 3' UTR of non-polyadenylated histone genes. (a) MEME analysis (Multiple Em for Motif Elicitation) [22,23] revealed the consensus sequence within the 3' UTR regions of histone genes for poly(A)- RNAs. (b) MFold analysis (version 3.5, M Zuker, Rensselaer Polytechnic Institute) predicted the stem-loop structure within the 3' UTR of histone genes for poly(A)- RNAs. [file gb-2011-12-2-r16-S11.PDF]
