# Supplementary material for: Genomewide characterization of non-polyadenylated RNAs
Source: Genome Biol. 2011 Feb 16;12(2):R16. doi: 10.1186/gb-2011-12-2-r16 (PMC3188798; doi:10.1186/gb-2011-12-2-r16)
Supplement: Additional file 18 — Gene-specific primers used for RT-PCR and qPCR validation. [file gb-2011-12-2-r16-S18.DOC]

**Additional file 18. Gene-specific primers for RT-PCR and real time RT-PCR validation.**

**A. Primer sequences used for RT-PCR validation.**

| Primer | Sequence | Description |
| --- | --- | --- |
| *rpph1*-F | CTGAGTGCGTCCTGTCACTC | pA- RNA |
| *rpph1*-R | GAGGAGAGTAGTCTGAATTGG |  |
| *terc*-F | TTTCTCGCTGACTTTCAGC | pA- RNA |
| *terc*-R | ACTCGCTCCGTTCCTCTTC |  |
| *h2afz*-F | GGTAAGGCTGGAAAGGACTC | pA+ histone mRNA |
| *h2afz*-R | GTAGCCTTGATGAGAGAATCC |  |
| *h2afx*-F1 | TCTACCTCGCTAGCATGTCG | Bimorphic histone mRNA |
| *h2afx*-R1 | AGCTTGTTGAGCTCCTCGTC |  |
| *h2afx*-R2 | AGTCTGAAGCGGCTCAGCTC |  |
| *h2be*-F | ATGCCTGAGCCAGCGAAATCC | pA- histone mRNA |
| *h2be*-R | CTCTGACACAGCGTGCTTGG |  |
| *h2bk*-F | TGACTAAGGCGCAGAAGAAG | pA- histone mRNA |
| *h2bk*-R | ATCTCCCTGGAGGTGATGGTC |  |
| *h3c*-F | GCAGCTTGCTACTAAAGCAG | pA- histone mRNA |
| *h3c*-R | CTTTGGGCATGATGGTGACG |  |
| *h1d*-F | TGCTCCTACCATTCCTGCAC | pA- histone mRNA |
| *h1d*-R | TTGGCCTTGGGTTTGCCTTC |  |
| *h3i*-F | GAGCTGCTAATCCGGAAGCTAC | pA- histone mRNA |
| *h3i*-R | GCTGATAGGAATATTTATGCCCTCTCC |  |
| *h3j*-F | CGTTATCAGAAGTCGACTGAG | pA- histone mRNA |
| *h3j*-R | GCAAGCTGGATGTCCTTAGG |  |
| *sno1*-F | CAGGAAGAGTTAGCCTGTAG | snoRNA precursor |
| *sno1*-R | GTGGCAGCCTATGTGTTCATGC |  |
| *malat1*-F1 | CCAGGTGCTACACAGAAGTG | pA- long ncRNA |
| *malat1*-R1 | CCTTCATCACCAAATTGCACTCG |  |
| *malat1*-F2 | GATGGTGTCGAGGTCTTTGG | pA- long ncRNA |
| *malat1*-R2 | CACTCAAATGCCTATCTTCTC |  |
| *ncl*-F | GATGATAGCAGTGGAGAAGAG | pA+ mRNA |
| *ncl*-R | AGCAGCTGCTGCTTTCATCG |  |
| *ubb*-F | GTGGACGTGGTTGGTGATTG | pA+ mRNA |
| *ubb*-R | GCTCCACCTCCAGAGTGATG |  |
| *oct3/4*-F | GACAGGGGGAGGGGAGGAGCTAGG | stem cell marker |
| *oct3/4*-R | CTTCCCTCCAACCAGTTGCCCCAAAC |  |
| l*in28*-F | GGGGAATCACCCTACAACCT | stem cell marker |
| *lin28*-R | GGGGAATCACCCTACAACCT |  |
| *hcgβ*-F | TGGCCTTGTCTACCTCTTGC | trophoblast diff. marker |
| *hcgβ*-R | GCCTCGTGTACCTGGCTTTA |  |

B. Primer sequences used for real time RT-PCR validation.

| Primer | Sequence | Description |
| --- | --- | --- |
| *rpph1*-F | CTAACAGGGCTCTCCCTGAG | pA- RNA |
| *rpph1*-R | CAGCCATTGAACTCACTTCG |  |
| *terc*-F | GCCTTCCACCGTTCATTCTA | pA- RNA |
| *terc*-R | TGACAGAGCCCAACTCTTCG |  |
| *malat1*-F | GGTCTTTGGTGGGTTGAACT | pA- long ncRNA |
| *malat1*-R | TTCCCACCCAGCATTACAGT |  |
| *h2afz*-F | GCCGTATTCATCGACACCTAA | pA+ histone mRNA |
| *h2afz*-R | TGCAAGTGACGAGGGGTAAT |  |
| *h2bk*-F | ACAAGGTGCTGAAGCAGGTC | pA- histone mRNA |
| *h2bk*-R | GGTCGAGCGCTTGTTGTAAT |  |
| *ccng1*-F | ATGGATTGTTTCTGGGCGTA | Bimorphic mRNA |
| *ccng1*-R | GGTTGTGGAGAAAGGCTTCA |  |
| *gprc5a*-F | TCCTCATGTCCTCCTTCACC | Bimorphic mRNA |
| *gprc5a*-R | GGAGCTGAGGATGGTGTCAT |  |
| *nr6a1*-F | CTCACCGTTTACAGCAAGCA | Bimorphic mRNA |
| *nr6a1*-R | TGCAAGCATACTCCTCGTTG |  |
| *ncl*-F | CCTCAGCAAAGAAGGTGGTC | pA+ mRNA |
| *ncl*-R | GCCAGGTGTGGTAACTGCTT |  |
| *pkr*-F | CTGTTGATGGCACTCTGGAA | pA+ mRNA |
| *pkr*-R | GGTCAATTGTGGGCTTCACT |  |
| *neat1*-F | TCGGGTATGCTGTTGTGAAA | pA+ long ncRNA |
| *neat1*-R | TGACGTAACAGAATTAGTTCTTACCA |  |
