# Supplementary material for: Genomewide characterization of non-polyadenylated RNAs
Source: Genome Biol. 2011 Feb 16;12(2):R16. doi: 10.1186/gb-2011-12-2-r16 (PMC3188798; doi:10.1186/gb-2011-12-2-r16)

Correlation among samples

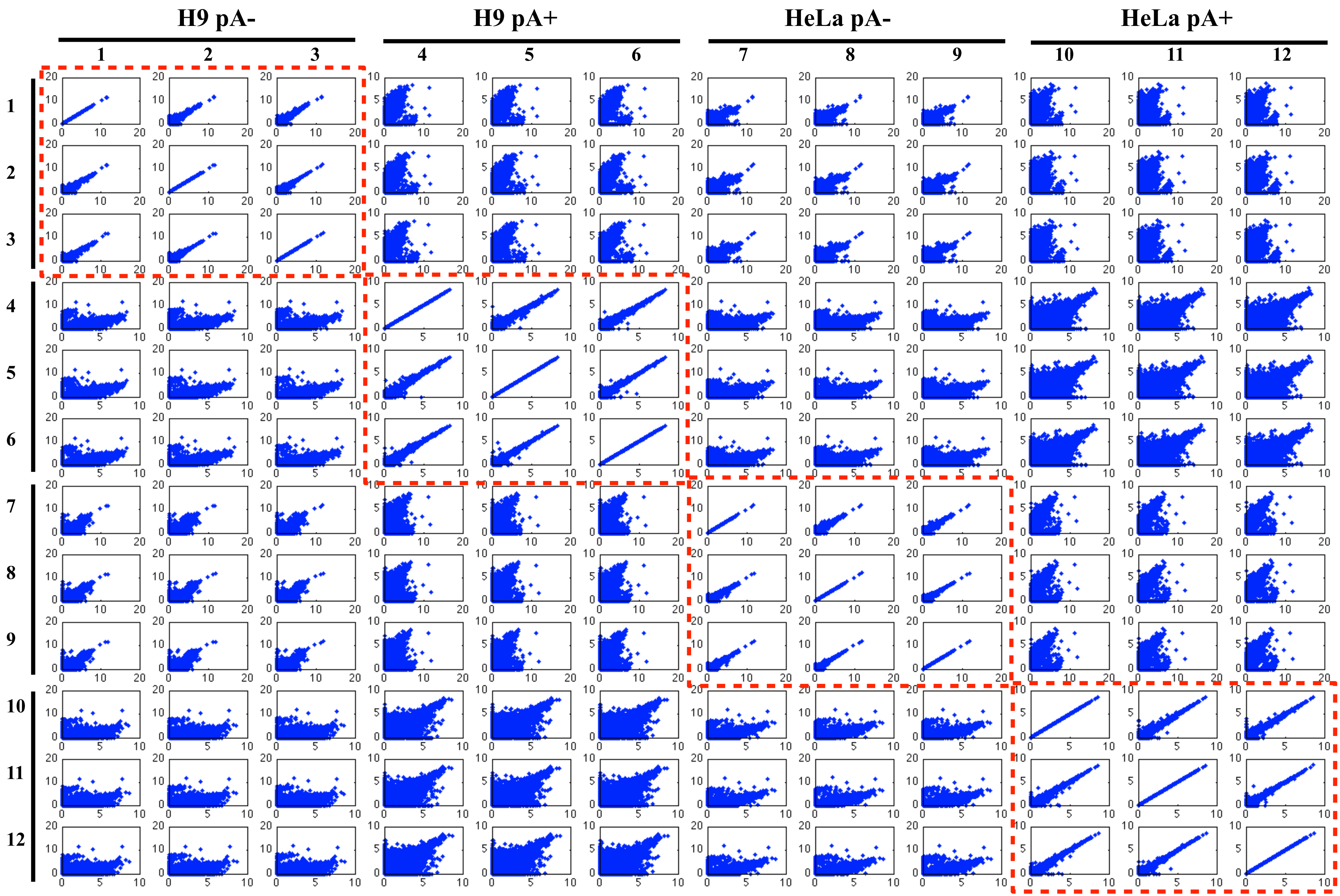

Supplement: Additional file 1 — Correlation among poly(A)+ and poly(A)- samples in H9 and HeLa cells. The scale for all plots is log10. 1-3, the sequencing triplicates of sample poly(A)- transcripts in H9 cells; 4-6, the sequencing triplicates of sample poly(A)+ transcripts in H9 cells; 7-9, the sequencing triplicates of sample poly(A)- transcripts in HeLa cells; 10-12, the sequencing triplicates of sample poly(A)+ transcripts in HeLa cells. Note the linear correlation among triplicates of individual samples is greater than 98%. [file gb-2011-12-2-r16-S1.PDF]
